# Infantile/Congenital High-Grade Gliomas: Molecular Features and Therapeutic Perspectives

**DOI:** 10.3390/diagnostics10090648

**Published:** 2020-08-28

**Authors:** Giulia Ceglie, Maria Vinci, Andrea Carai, Sabrina Rossi, Giovanna Stefania Colafati, Antonella Cacchione, Assunta Tornesello, Evelina Miele, Franco Locatelli, Angela Mastronuzzi

**Affiliations:** 1 Department of Onco-Hematology and Cell and Gene Therapy, Bambino Gesù Children’s Hospital (IRCCS), Piazza Sant’Onofrio 4, 00146 Rome, Italy; maria.vinci@opbg.net (M.V.); antonella.cacchione@opbg.net (A.C.); evelina.miele@opbg.net (E.M.); franco.locatelli@opbg.net (F.L.); 2 Neurosurgery Unit, Department of Neurological and Psychiatric Sciences, Bambino Gesù Children’s Hospital (IRCCS), Piazza Sant’Onofrio 4, 00146 Rome, Italy; andrea.carai@opbg.net; 3 Pathology Unit, Department of Laboratories, Bambino Gesù Children’s Hospital (IRCCS), Piazza Sant’Onofrio 4, 00146 Rome, Italy; sabrina2.rossi@opbg.net; 4 Neuroradiology Unit, Department of Imaging, Bambino Gesù Children’s Hospital (IRCCS), Piazza Sant’Onofrio 4, 00146 Rome, Italy; gstefania.colafati@opbg.net; 5 Pediatric Oncology Unit, Ospedale Vito Fazzi, Piazza Filippo Muratore, 1, 73100 Lecce, Italy; assuntatornesello@gmail.com; 6 Department of Maternal, Infantile, and Urological Sciences, University of Rome La Sapienza, Piazzale Aldo Moro 5, 00185 Rome, Italy

**Keywords:** brain tumors, high-grade gliomas, congenital cancer, neuro-oncology, neonatal cancer, neurotrophic tyrosine receptor kinase

## Abstract

Brain tumors in infants account for less than 10% of all pediatric nervous system tumors. They include tumors diagnosed in fetal age, neonatal age and in the first years of life. Among these, high-grade gliomas (HGGs) are a specific entity with a paradoxical clinical course that sets them apart from their pediatric and adult counterparts. Currently, surgery represents the main therapeutic strategy in the management of these tumors. Chemotherapy does not have a well-defined role whilst radiotherapy is rarely performed, considering its late effects. Information about molecular characterization is still limited, but it could represent a new fundamental tool in the therapeutic perspective of these tumors. Chimeric proteins derived from the fusion of several genes with neurotrophic tyrosine receptor kinase mutations have been described in high-grade gliomas in infants as well as in neonatal age and the recent discovery of targeted drugs may change the long-term prognosis of these tumors, along with other target-driven therapies. The aim of this mini review is to highlight the recent advances in the diagnosis and treatment of high-grade gliomas in infants with a particular focus on the molecular landscape of these neoplasms and future clinical applications.

## 1. Introduction

Brain tumors in infants are rare entities and their definition is still debated. Most authors define them as brain tumors occurring in children in the first years of life, thus including tumors diagnosed during fetal development, neonatal age and under the age of one year [1]. Other authors consider “brain tumors in infancy” as tumors diagnosed until three to five years of age defining a continuous category of infants and very young patients [2]. Infant brain tumors account for around 10% of all pediatric brain tumors [1] and around half of them (5%) occur in the first six months of life [2]. In this review, we have focused on children under one year old as most statistics present in medical literature use this limit for the definition of infantile tumors. Where the statistics refer to a different age group this has been specified in the text.

Congenital brain tumors are a subgroup whose definition is still unclear. Some authors [3] divide them into “definitely congenital” (evident or symptomatic at birth), “probably congenital” (evident or symptomatic within the first week of life) and “possibly congenital” (present or producing symptoms within the first six months of life) [4].

The rarity of infantile brain tumors poses a significant challenge to the clinician. Firstly, they are not easy to diagnose as the flexibility of neonatal skull leads to a delayed symptomatic response to the increased intracranial pressure and secondly, they are difficult to treat, representing a dilemma for the neuro-oncologist. This is in part due to the different histological characteristics [5] and in part to the objective difficulty in treating such a population where all approaches are a potential risk: surgery, with the attendant anesthesiologic risk and chemotherapy and radiotherapy, because of the long-term complications.

As for congenital tumors, teratoma is the most frequent, accounting for about one third to one half of all cases, followed by glioma and choroid plexus papilloma accounting for 18–47% and 5–20% of all perinatal brain tumors, respectively [3].

Gestational age can orient towards a specific histological diagnosis; for example, tumors arising before the 22nd week are often teratomas or hamartomas. Between weeks 22 and 32, germ cell tumors are frequently observed; after the 32nd week, astrocytomas and glioblastomas are detected [6].

Among all infantile brain tumors, in various studies [5,7] the most common histological diagnosis is reported to be glioma, with low-grade gliomas being more common. The next most common diagnosis is medulloblastoma, followed by ependymoma [8].

In this setting, infantile gliomas are neoplasms with characteristics that make them different from other infantile brain tumors and their counterparts in older children and adults [9]. In the last decades, advances in the molecular characterization of tumors have widely contributed to our understanding of the molecular basis of these tumors, making a tremendous difference not only in their characterization, but also in the therapeutic armamentarium at our disposal.

The aim of this review is to summarize the current knowledge of infantile high-grade gliomas (iHGGs) and to examine the recent advances in diagnosis and treatment.

## 2. Materials and Methods

The authors conducted a literature search describing the issue of infant HGGs. The search terms used were congenital brain tumors, neonatal brain tumors, infantile brain tumors and fetal brain tumors. Only papers written in English were considered and those published from the year 2000 up to May 2020 were mostly selected. We included reviews, case series and research studies that were classified according to their relevance. No abstracts were included.

## 3. Clinical Findings and Diagnosis

We have discussed in the introduction how the definition of infantile tumors, specifically the clinical characteristics of iHGGs, is still a debated issue. To help the reader navigate into these peculiarities, in the following section we have outlined the essential notions to approach the clinical findings and diagnostic tools of these neoplasms. 

As known, the fontanelles allow for stretching and deformation of the head as the brain expands faster than the surrounding bone. This flexibility often leads to a delay in the onset of symptoms as the infant skull is capable of accommodating the increasing intracranial pressure and when symptoms do arise they are often nonspecific [10]. In the pediatric population, the most common symptoms of a brain tumor are headache and early morning vomiting. Other symptoms can present depending on tumor localization; for example, disconjugate gaze and ataxia are frequently encountered in patients with infratentorial tumors. On the other hand, seizures, disturbed vision and other focal neurological signs are seen more commonly in patients with supratentorial tumors [10]. As for tumor location, infant brain tumors are more often supratentorial than those diagnosed later in childhood [11]. However, all the symptomatic findings enumerated above are not easy to interpret in very young children. For the infant population, we often have to rely on other, more subtle and non-specific signs and symptoms. In the antenatal period, polyhydramnios is the most common presentation, observed in approximately one third of patients. It is caused by depressed swallowing due to hypothalamic dysfunction and may be the first clinical sign noted during obstetric examination [12]. Another very common feature is macrocephaly, which can be a consequence of the intracranial expansion of the tumor or hydrocephalus. Hydrocephalus, in turn, is most commonly caused by an obstruction of the ventricular system, but it can also arise from increased cerebrospinal fluid production by a choroid plexus tumor. These anomalies can be detected by pre-natal ultrasound and they are most commonly encountered in the third trimester [13]. In these cases, fetal Magnetic Resonance Imaging (MRI) can be of help in confirming these findings, but it is rarely helpful in distinguishing between individual tumor types. Some of these tumors grow enormously during the pregnancy and may cause stillbirth, which is particularly associated with intracranial teratomas, glioblastomas and primitive neuroectodermal tumors (PNETs) [14]. Brain tumors diagnosed during gestation often require a cesarean section as neonates born by vaginal delivery often develop dystocia as a consequence of a difficult labor. Macrocephaly is also the earliest post-natal sign of a neonatal brain tumor; this may be associated with bulging fontanelles caused by the delayed fusion of the anterior fontanelle (normally fused approximately by 12 months) [15]. Later in infancy, other symptoms may arise such as a failure to thrive, apneic episodes, irritability, delay in developmental milestones, drowsiness, irritability, seizures, somnolence, vomiting and abnormal eye movements [3]. All of these symptoms are non-specific and much more difficult to interpret in the neonate than in an older child. As described in the retrospective cohort by Toescu et al., the most common presenting symptom in the first year of life is vomiting and the most common clinical sign is an augmented head circumference, followed by a bulging fontanelle [16].

Associated congenital anomalies are in some cases indicative of congenital tumors such as cleft lip or cleft palate and malformations of the heart and urinary tract. Cleft palate/lip is associated with teratomas and low-set ears are associated with craniopharyngiomas [15]. Sometimes the identification of a brain tumor and an intracardiac mass, such as a rhabdomyoma, could suggest a genetic syndrome such as the tuberous sclerosis complex (TSC) [17], indicating the need for genetic counseling and testing. The main differential diagnosis for an intracranial tumor is hemorrhage, which may also manifest as a disorganized intracranial mass and/or hydrocephalus. On the other hand, congenital brain tumors have a propensity to bleed and intratumoral bleeding is frequently observed. Therefore, in the case of a spontaneous intracranial hemorrhage, an underlying neoplasm should always be excluded [3].

Biopsy and histopathological examinations are essential for a definitive diagnosis of a brain tumor. They can be very difficult to obtain in infants and it is virtually impossible to obtain a fetal biopsy specimen safely [14]. Therefore, imaging has a fundamental role in the diagnosis of these tumors.

Cranial ultrasonography (US) and MRI are the mainstays of diagnostic evaluation. With US, most intracranial tumors have a heterogeneous pattern with the subversion of normal structures. In particular, teratomas are usually associated with calcifications, which may be important for diagnosis. If there is the suspicion of a tumor, MRI should be the next step. This technique allows for a detailed assessment of tumor morphology and its spatial relationships with the surrounding structures; essential information for an eventual surgical approach [18]. The main disadvantage of MRI is that the imaging procedure takes a long time and during acquisition the patient must remain perfectly still. This difficulty may be overcome with sedation, thus exposing the child to an anesthesiologic risk. New strategies for immobilizing the child for several minutes without anesthesia are being explored; one being to perform the scan just after the neonate has fed and is at his most drowsy, another is using infant incubators/immobilizers or sucrose solutions [19]. 

Another disadvantage of MRI is its inadequacy in identifying the calcifications that are characteristic of some histological subtypes such as oligodendrogliomas and gangliogliomas. Computed tomography (CT) is more useful to identify calcifications but exposes the patient to a large dose of ionizing radiation [18].

## 4. Neuropathology and Molecular Characteristics of iHGGs

As stated before, gliomas are reported as the most common infant tumor histology, with a predominance of low-grade features. iHGGs can show certain common histopathological features and are often driven by distinct gene fusions.

Some studies have highlighted peculiar histopathological features in infant HGG regarding the pattern of growth, i.e., the sharp demarcation between the tumoral and non-tumoral tissue with no evidence of infiltration of the surrounding CNS tissue as well as the cytomorphology, which appears typically monomorphous and often features either minigemistocytes or spindle cells [20,21]. It is unlikely that these morphological characteristics are per se sufficient to explain the paradoxical clinical course of infant HGGs. However, it may be speculated that this somewhat typical morphology, along with the specific DNA methylation profiling and the genetic signature described in the following paragraph, mirror a different cell of origin of congenital HGG compared with the adult counterpart.

The most common somatic alterations found in iHGGs seem to be gene fusions involving *NTRK1/2/3* (neurotrophin receptor tyrosine kinase) genes, which encode for tropomyosin receptor kinase (Trk) A, B and C, respectively. These alterations have been described in pediatric HGG and in particular in 40% of non-brainstem HGGs in infants [22,23,24,25]. All fusions include the C-terminal kinase domain from NTRK1, NTRK2 or NTRK3 with the N-terminal sequences of different genes, leading to the transcription of chimeric Trk proteins with constitutively activated or overexpressed kinase function and subsequent oncogenic potential. Other gene fusion alterations found in iHGGs are *AGBL4:NTRK2, TPM3:NTRK1* and *ETV6:NTRK3* [22]. *NTRK* gene fusions are emerging as novel targets not only for iHGGs but also across multiple tumor types due to the growing availability of new drugs with anti-Trk activity [22].

Congenital glioblastoma (cGBM) is among the rarest type of congenital brain tumor [21]. Children with these tumors can be stillborn or have an extremely poor prognosis of approximately two months among untreated patients [26]. This very poor prognosis might in part be due to the tendency of bleeding and intracranial hemorrhage; on the other hand, in the absence of these complications, patients seem to have a more favorable prognosis following limited or no treatment [22,27]. These observations indicate that cGBMs may have a more unpredictable and maybe favorable outcome than those in older children and adults. As for molecular alterations, in adults the most frequent alterations involve the epidermal growth factor receptor (*EGFR*), platelet-derived growth factor receptor (*PDGFR*), phosphatase and tensin homolog (*PTEN*), *INK4a/ARF* and *TP53* or, more rarely, Isocitrate dehydrogenase1 (*IDH1*) and *ATRX* [28]. In cGBM, the expression of EGFR and PDGFRa is low with a rare occurrence of copy number alterations in these genes [27,29]. In addition, while mutations in *TP53* and *PTEN* and *CDKN2A/B* deletions are often seen in older children they are typically not found infant HGG [30]. Even *IDH1* mutations are rare while occasional *BRAF* (B-Raf proto-oncogene, serine/threonine kinase) V600E mutations can be found [31].

Gene expression profile has identified 31 differentially expressed genes in cGBMs compared with pediatric non-congenital glioblastoma (pGBMs) and primary adult GBMs (aGBMs) [21]. In agreement with previous findings [32] and as already described in pGBMs [33], no amplification of *EGFR* as measured by fluorescence in situ hybridization (FISH) was observed in cGBM samples and *EGFR* gene expression was very low. Similarly, no PDGFRa amplification was seen by FISH in cGBMs and *PDGFRa* gene expression levels were much lower than those seen in a subset of patients with pGBM, suggesting that the amplification of *PDGFRa* may be unusual in cGBMs, unlike pGBMs [34]. No down-regulation of phosphatase and tensin homolog (*PTEN*) or up-regulation of *MYCN* proto-oncogene, *BHLH* transcription factor (*MYCN*) was suggested from the cGBM gene expression data. On the contrary, *EGFR* and *PDGFRa* alterations, including amplification or mutations, have been reported in 57% and 13% of aGBMs, respectively, as well as frequent *PTEN* mutations/deletions and gains of *MYCN* [30]. None of the patients with cGBMs had the recently described histone H3.3 mutations found in pGBMs, which are instead extremely rare in aGBMs [35]. Finally, functional analysis revealed that 50% of the differing genes were involved in signal transduction whilst 39% were involved in glucose metabolism. Notably, derangements in another metabolic pathway involved in energy production have been described including glutamate homeostasis through the aberrant expression of the mitochondrial enzyme *GLUD2* in glioblastoma tumor cells [36,37].

Recently, DNA methylation profiling has been introduced to improve molecular classification of brain tumors, exploiting the notion that the cancer methylome is a combination of both somatically acquired DNA methylation changes and features resembling the tumor cell of origin [38].

In a recent report [39], DNA methylation and transcriptomic analysis showed a lower incidence in cGBMs compared with pGBMs and aGBMs of typical GBM-related copy number aberrations (CNAs) but identified cyclin dependent kinase 6 (*CDK6*) and cyclin dependent kinase inhibitor 2A/B (*CDKN2A/B*) deletions and MET proto-oncogene, a receptor tyrosine kinase (*MET*) fusion gene. Again, no amplification of *EGFR, PDGFRa* and *MYCN* or *PTEN* loss was observed. In both studies [21,39], cGBMs were associated with a better prognosis than pediatric or adult GBMs, which could be the result of the identified gene expression differences.

In addition to these findings, Gielen et al. described a loss of small nucleolar RNA and C/D box (*SNORD*) genes encoding for C/D box snoRNAs on chromosome 2 and chromosome 14 in infants with GBMs [29]. snoRNAs are considered to play a crucial role in post-transcriptional modifications of target RNAs and their loss may represent a driving oncogenic event in tumor development.

In this wide field of genetic characterization, epigenetic analysis has contributed to our understanding of the recurrent molecular pattern of iHGGs, mainly thanks to studies of methylation profiling that have recently emerged. These studies have shown that iHGGs may display a more LGG-like methylation pattern, with a two year survival of 74% [40]. A recent international collaborative study investigated methylation and gene expression data in this rare subgroup of patients [23]. The authors identified distinct subgroups with an ‘intrinsic’ spectrum of disease specific to the infant population. One group was characterized by MAP-kinase alterations, while a second one comprised cases harboring gene fusions targeting *NTRK1/2/3, ALK* and less common *ROS1* and *MET* as their driving alterations. Beside *NTRK1/2/3*, as described above, *ALK* gene fusions included several partner genes, some of which have been previously described including *PPP1CB* (the most common), *EML4, HIP1, PRKAR2A, SPTBN1* [24,25,41,42] and novel genes such as *MAD1L1, MAP2, MSI2, SPECC1L1, SYNDIG1L, ZC3H7A* and *CLIP2* [43].

These alterations are targetable lesions with evidence of efficacy in the clinic, thus supporting the concept that infant gliomas require a change in diagnostic practice and management.

*BRAF* V600E mutation has been recently detected in 17% of patients with pediatric low-grade gliomas (including infants) and in HGGs [44]. *BRAF* V600E confers worse outcomes when patients are treated with chemotherapy and radiation compared with *BRAF* wild-type patients, especially when *CDKN2A* deletion [45] occurs. The same group published a case report on a two month old patient with a V600E mutant hypothalamic/chiasmatic glioma who was successfully treated with the BRAF inhibitor dabrafenib, which resulted in a rapid and sustained disappearance of clinical symptoms and cytoreduction [46]. In addition, several reports have been published of pediatric LGGs harboring the *BRAF* V600E mutation that was successfully treated with Vemurafenib [47,48]. Since this mutation has also been described in the infant population, a *BRAF*-inhibitor might also have a role in the treatment of this rare subgroup of HGGs.

## 5. Treatment Options

As the group of infantile brain tumors is extremely heterogeneous, comprising entirely different entities, it is not easy to navigate through all the actual therapeutic options. In this small section, we will try to give the reader the basic principles for the treatment of these neoplasms. The discussion will only focus on iHGGs.

The principal treatment for iHGGs is surgery aiming for a gross total resection. The extent of resection of surgery directly correlates with prognosis as the extent of surgical resection is correlated with the overall outcome [49,50]. Moreover, surgery also offers histological specimens allowing a more accurate diagnosis. The two principal modalities available to treat CNS tumors postoperatively are chemotherapy and radiotherapy. Craniospinal irradiation used to be the mainstay treatment for childhood CNS tumors. However, its use was limited by the serious sequelae, such as developmental delay, endocrine dysfunction and secondary neoplasms in the CNS especially in children under the age of three. In this age group, radiation therapy was not only associated with more severe side effects, but also with less improvement in outcome [18]. In this context, studies have demonstrated higher long-term survival rates without radiotherapy of infant patients with HGG when compared with other tumor histologies [51].

As for Chemotherapy, the chemotherapeutic drugs mainly used in the treatment of HGGs in very young children (<10 years old) are vincristine, carboplatin, temozolomide and thiotepa [52].

Since the 1980s, several chemotherapy regimens have been examined in the early “baby brain studies” for all types of brain tumors in very young children. Several chemotherapeutic agents were tried in different combinations to be administered for long periods in order to replace or postpone radiotherapy [53,54] and many of these trials demonstrated the effectiveness of chemotherapy alone to sustain long-term response and survival.

In addition, the role of high-dose chemotherapy followed by autologous stem cell rescue has been investigated in very young children with HGG [55].

Considering the limited therapeutic strategies in these patients and the recent widened landscape of the molecular alterations previously described, novel targeted agents are being validated.

As previously described, the most common somatic alterations found in infantile HGG are TRK fusions that have a critical role in tumorigenesis in 40% of infant HGGs [41]. Larotrectinib is the first selective TRK inhibitor, active on all TRK proteins, in clinical development. This highly selective small molecule has shown activity in preclinical models and in adults with tumors harboring TRK fusions [55,56]. Recently, it has been demonstrated that the agent is also well tolerated in pediatric patients and showed encouraging antitumor activity in patients with TRK fusion-positive tumors [56]. The pediatric dose was defined as 100 mg/m^2^ (maximum 100 mg per dose) for infants, children and adolescents, regardless of age [57]. Subsequent clinical trials confirmed the anti-tumor activity of larotrectinib in the pediatric population [57] even though the characterization of its safety profile, especially regarding its delayed consequences and its protracted use, are still under monitoring in the pediatric population where it may pose the biggest concerns [58]. The ongoing NCT02576431 and NCT02637687 trials will further increase the number of patients studied in disease-specific settings and may help to monitor its safety and efficacy data.

Another group of targetable genetic lesions that may be exploited for the treatment of iHGGs is ALK alteration [23,24,25]. The availability of ALK inhibitors introduces the possibility of using these targeted agents in the treatment of these patients. In particular, second generation ALK inhibitors such as ceritinib, alectinib, brigatinib and ensartinib have shown efficacy especially in the central nervous system (CNS) [59]. Lorlatinib, the new ALK and ROS1 inhibitor, fits into the context as it has demonstrated substantial CNS activity due to its ability to penetrate the blood-brain barrier [60]. The efficacy and safety of these agents have also been described and evaluated in the pediatric context, hopefully making it a feasible option also in infants in the near future [61].

**Figure 1:**
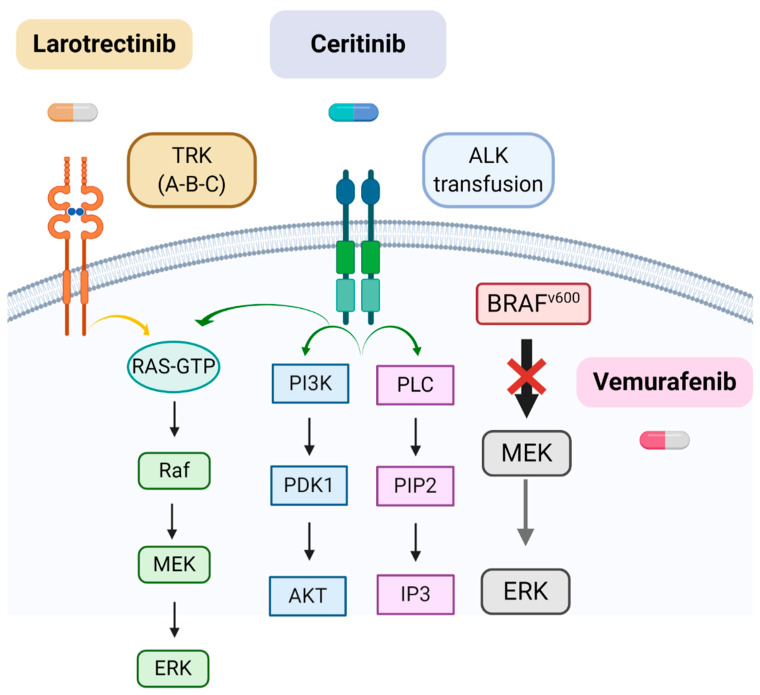
Main targetable molecular alterations in iHGGs. Note that the drug Vemurafenib acts blocking only the mutated form of *BRAFV*. Created with Biorender.com.

The following table (Table 1) and Figure (Figure 1) summarize the main molecular alterations found in iHGGs and other characteristics discussed in the text.

## 6. Conclusions

Infantile brain tumors are a rare entity in pediatric oncology and their characterization is far from being complete. In this article, we summarized the current knowledge about HGGs in infancy and provided an up-to-date review of the main characteristics with special attention paid to the molecular landscapes. It is known that these tumors have a different clinical behavior when compared between their pediatric and adult counterparts; moreover, in the pediatric population, surgery and radical resection represent the main curative options since chemotherapy and radiation therapy have significant long-term toxicities that makes them a limited option in such a young group of patients. In this setting, it is of utmost importance to improve the molecular characterization in order to identify new targets for the possible identification of new biology-driven therapeutic approaches. As we have described, the precise molecular characterization of these tumors has made it possible not only to increase our knowledge to better understand the different prognosis of these patients, but also to explore new targeted therapies. In the context of such a young population, the possibility of sparing aggressive surgical, chemotherapeutic or radiotherapy treatment is extremely important as neurological sequelae are very severe. On the other hand, the vast majority of these novel agents have only been recently introduced to the pediatric population therefore the experience of these is still limited. Several years and hopefully decades of follow-up will increase our knowledge of the long-term effects of these new targeted agents.

## Figures and Tables

**Table 1:** Main targetable molecular alterations in infantile high-grade gliomas (iHGGs).

Molecular Alteration	Type of iHGGs	Target Therapy	Refs
*TRK* fusions (*NTRK1/2/3*)	Non-brainstem iHGGs	Larotrectinib	[56,57,58]
*ALK* rearrangements	IHG and LGG-like DIGG/DIA and IHG	Ceritinib, Alectinib, Brigatinib and Ensartinib	[55,59]
*BRAF* V600E	Hypothalamic/chiasmatic glioma	Vemurafenib	[46,47,48]
